# Abnormal increase in urinary aquaporin-2 excretion in response to hypertonic saline in essential hypertension

**DOI:** 10.1186/1471-2369-13-15

**Published:** 2012-03-27

**Authors:** Carolina Cannillo Graffe, Jesper Nørgaard Bech, Thomas Guldager Lauridsen, Henrik Vase, Erling Bjerregaard Pedersen

**Affiliations:** 1 Department of Medical Research, Holstebro Hospital, Laegaardvej 12, 7500 Holstebro, Denmark; 2 Department of Medicine, Holstebro Hospital, Holstebro, Denmark; 3 University of Aarhus, Aarhus, Denmark

## Abstract

**Background:**

Dysregulation of the expression/shuttling of the aquaporin-2 water channel (AQP2) and the epithelial sodium channel (ENaC) in renal collecting duct principal cells has been found in animal models of hypertension. We tested whether a similar dysregulation exists in essential hypertension.

**Methods:**

We measured urinary excretion of AQP2 and ENaC β-subunit corrected for creatinine (u-AQP2_CR_, u-ENaC_β-CR_), prostaglandin E2 (u-PGE_2_) and cyclic AMP (u-cAMP), fractional sodium excretion (FE_Na_), free water clearance (C_H2O_), as well as plasma concentrations of vasopressin (AVP), renin (PRC), angiotensin II (Ang II), aldosterone (Aldo), and atrial and brain natriuretic peptide (ANP, BNP) in 21 patients with essential hypertension and 20 normotensive controls during 24-h urine collection (baseline), and after hypertonic saline infusion on a 4-day high sodium (HS) diet (300 mmol sodium/day) and a 4-day low sodium (LS) diet (30 mmol sodium/day).

**Results:**

At baseline, no differences in u-AQP2_CR _or u-ENaC_β-CR _were measured between patients and controls. U-AQP2_CR _increased significantly more after saline in patients than controls, whereas u-ENaC_β-CR _increased similarly. The saline caused exaggerated natriuretic increases in patients during HS intake. Neither baseline levels of u-PGE_2_, u-cAMP, AVP, PRC, Ang II, Aldo, ANP, and BNP nor changes after saline could explain the abnormal u-AQP2_CR _response.

**Conclusions:**

No differences were found in u-AQP2_CR _and u-ENaC_β-CR _between patients and controls at baseline. However, in response to saline, u-AQP2_CR _was abnormally increased in patients, whereas the u-ENaC_β-CR _response was normal. The mechanism behind the abnormal AQP2 regulation is not clarified, but it does not seem to be AVP-dependent.

**Clinicaltrial.gov identifier:**

NCT00345124.

## Background

Dysregulation of the expression/shuttling of the renal epithelial sodium channel (ENaC) and the aquaporin-2 water channel (AQP2) has been suggested to play a role in the pathogenesis of essential hypertension.

ENaC is responsible for the reabsorption of sodium through the apical membrane of the connecting tubule and the collecting duct and plays a key role in controlling sodium balance, extracellular fluid volume, and blood pressure. ENaC is a heteromultimeric protein composed of three homologous subunits (α, β and γ) [1,2]. Aldosterone (Aldo) is the main hormonal regulator of ENaC [3,4]. Binding of Aldo to the intracellular mineralocorticoid receptor increases the transcription and the apical translocation of ENaC [5,6]. ENaC is excreted into the urine. Recently, our group demonstrated a significant correlation between changes in the urinary excretion of the ENaC β-subunit (u-ENaC_β_) and changes in urinary sodium excretion [7]. Thus, u-ENaC_β _has been suggested as a marker of the transport of sodium via ENaC.

AQP2 is the apical water channel of collecting duct principal cells. AVP is the main hormonal regulator of AQP2 [8,9]. Binding of AVP to V2 receptors in the basolateral membrane stimulates adenylate cyclase producing cAMP and protein kinase A (PKA). Short-term AVP exposure results in trafficking of subapical vesicles containing AQP2 to the apical plasma membrane, whereas long-term exposure causes a marked increase in the AQP2 whole-cell abundance via regulation of AQP2 gene transcription and AQP2 protein degradation [9-12]. Withdrawal of AVP leads to retrieval of AQP2 from the apical plasma membrane into subapical vesicles [11]. AQP2 is excreted into the urine [13-15] and is used as a marker for the action of AVP on the collecting ducts.

An increased expression of ENaC subunits and an increased expression and apical targeting of AQP2 has been reported in spontaneous hypertensive rats, an experimental model of hypertension [16-18]. These results adjoined with the existence of 1) an abnormal natriuresis in essential hypertension, 2) an abnormal pressure-natriuresis relationship in essential hypertension, 3) a genetic linkage between a monogenic form of human hypertension, Liddle's syndrome, and ENaC [19], 4) a genetic linkage between systolic blood pressure and ENaC subunits in essential hypertension [20-22], and 5) an association of ENaC polymorphisms and essential hypertension [23-25], suggest that an abnormal regulation of the expression/shuttling of AQP2 and/or ENaC could be involved in the pathogenesis of essential hypertension.

In the present study, we wanted to test the hypothesis that u-AQP2 and u-ENaC were abnormal in essential hypertension during dietary high sodium (HS) intake and/or during dietary low sodium (LS) intake, and that these variables responded abnormally to a hypertonic saline infusion.

In order to analyse the regulation of the expression/shuttling of AQP2 and ENaC in essential hypertension, we performed a randomised, cross-over trial with patients with essential hypertension and normotensive control subjects. We compared the absolute values of u-AQP2 and u-ENaC_β _corrected for creatinine (u-AQP2_CR _and u-ENaC_β-CR_), fractional sodium excretion (FE_Na_), free water clearance (C_H2O_), urinary excretion of prostaglandin E2 (u-PGE_2_), urinary excretion of cyclic-AMP (u-cAMP), and plasma concentrations of AVP, renin (PRC), angiotensin II (Ang II), Aldo, atrial natriuretic peptide (ANP), and brain natriuretic peptide (BNP) in patients with essential hypertension and normotensive controls during both HS and LS intake. Furthermore, we compared the relative changes in the above mentioned effect variables after a hypertonic saline infusion during both diets.

## Methods

### Patients and control subjects

#### Patients

*The inclusion criteria *for patients with essential hypertension were: 1) white men and women, 2) age between 18 and 65 years, 3) arterial hypertension, defined by 24-h ambulatory blood pressure above 125 mmHg systolic or 80 mmHg diastolic, and 4) body mass index (BMI) ≤ 30 kg m^-2^. *The exclusion criteria *were: 1) abnormal renography, 2) a medical history or clinical signs of heart, lung, liver, kidney, brain, endocrine organ, cardiovascular or neoplastic disease, 3) severe hypertension, 4) abnormal biochemical screening of the blood regarding haemoglobin, white blood cell count, platelets, sodium, potassium, creatinine (> 200 μmol l^-1^), albumin, bilirubin, alanine aminotransferase, alkaline phosphatase, cholesterol, and glucose, 5) abnormal urine screening for blood, albumin, and glucose, 6) abnormal electrocardiogram, 7) drug or alcohol abuse, 8) smoking, 9) pregnancy or breast feeding, 10) blood donation less than one month before the examination, and 11) unwillingness to use contraceptives if fertile woman (in order to avoid to infuse potentially pregnant women with 51Cr-EDTA and hypertonic saline). *The withdrawal criteria *were: 1) lack of compliance, 2) withdrawal of consent, and 3) development of one of the exclusion criteria during the study. Antihypertensive agents were discontinued two weeks before each study day. The blood pressure of the subjects was controlled once a week in the two week period. If blood pressures rose to levels above 170 mmHg systolic or 105 mmHg diastolic, substitution treatment with metoprolol was initiated (this did not happen in any of the patients).

#### Control subjects

*The inclusion criteria *for the normotensive controls were: 1) white men and women, 2) age between 18 and 65 years, 3) 24-h ambulatory blood pressure below 125 mmHg systolic and 80 mmHg diastolic. *The exclusion- and withdrawal criteria *were the same as for the patients. None of the normotensive controls received any medication, except oral contraceptives.

### Recruitment

The patients with essential hypertension were recruited from the Nephrological out-patient clinic of the Department of Medicine, Holstebro Hospital. The control subjects were recruited by advertising in public institutions and private companies.

### Ethics and approvals

The study was approved by the local Medical Ethics Committee (JRN RRS-2006-1014-(2733-06)). The study was conducted in conformity with the principles of the Declaration of Helsinki, and written informed consents were obtained from all the subjects. The study was registered at the registration site: http://www.clinicaltrials.gov (NCT00345124).

### Design

We performed two randomized, cross-over studies, one with patients with essential hypertension and one with normotensive controls. Each subject was studied on two separate days at least three weeks apart. During four days before the study day, the subjects consumed either a HS or LS diet in randomized order. The results of the patients are compared to the results of the normotensive controls. Furthermore, in both patients and controls the results obtained during HS and LS intake are compared.

### Effect variables

The primary effect variable was u-AQP2_CR_, and the secondary effect variables were urinary sodium excretion rate (U_Na_), FE_Na_, u-ENaC_β-CR_, urine volume (V), C_H20_, serum osmolality (s-osm), urine osmolality (u-osm), u-cAMP and u-PGE_2_, plasma concentrations of AVP, PRC, Ang II, Aldo, ANP, BNP, sodium (p-Na), and albumin (p-albumin), systolic and diastolic blood pressure, heart rate, body weight, and glomerular filtration rate (GFR).

### Number of subjects

A difference in u-AQP2 of 0.25 ng min^-1 ^was considered the minimal relevant difference based on interventions in previous pilot experiments. A sample size of 20-21 subjects who could be evaluated had 90% power to detect this difference assuming a level of significance of 5% and a standard deviation of 0.24 ng min^-1^. Because a few subjects were expected to drop out, 25 subjects were included in each group.

### Experimental procedure prior to the study day

Five days before the study day, the subjects collected a standardized, HS (approx. 300 mmol sodium day^-1^/17.5 g salt day^-1^) or LS (approx. 30 mmol sodium day^-1^/1.8 g salt day^-1^), 4-day diet from the hospital kitchen. Depending on the individually estimated energy requirement, the participants were given either a diet of 8,000 or 11,000 kJ day^-1^. The energy distribution was: 55% carbohydrates, 15% proteins, and 30% lipids. The 4-day diet was started the following morning.

The fluid intake was also standardized during the four days. The subjects were asked to drink exactly 250 ml per 1000 kJ day^-1 ^and to abstain from coffee, tea, and alcoholic beverages.

The subjects were instructed to keep their physical activity unchanged during the two experiments and to abstain from hard training.

The subjects collected their urine for 24 hours the day before the study day.

### Experimental procedure on the study day

On the study day, the subjects were asked to drink 175 ml of water every 30 minutes from 7:00 AM. The subjects arrived at the department at 8:00 AM. Peripheral IV lines were inserted into the antecubital veins of both forearms, one for infusion of 51Cr-EDTA and hypertonic saline, and one for withdrawal of blood samples. The subjects were kept in the supine position from 8:00 AM to 1:30 PM except during voiding, which took place in the sitting or standing position.

At 8:30 AM, a priming dose of 51Cr-EDTA was administered, followed by sustained infusion. After 60 minutes of equilibration, the study continued with five clearance periods, the first two of 30 minutes duration (P1-P2), the last three of 60 minutes duration (P3-P5). The first two clearance periods were baseline periods.

At 10:30 AM, 7 ml kg^-1 ^of 3% saline were given over 30 minutes.

Blood pressure and heart rate were measured every 30 minutes from 9:30 AM to 1:30 PM.

Urine was collected in each clearance period and analyzed for sodium, osmolality, u-AQP2, u-ENaC_β_, u-cAMP, u-PGE_2_, and 51Cr-EDTA.

Blood samples were drawn every 30 minutes from 9:30 AM to 10:30 AM and every hour from 11:30 AM to 1:30 PM, and were analyzed for sodium, osmolality, and 51Cr-EDTA. In addition, analysis of AVP, PRC, Ang II, Aldo, ANP and BNP were performed from blood samples drawn at 10:30 AM, 11:30 AM, 12:30 PM, and 1:30 PM.

### Methods

All blood samples were centrifuged for 15 minutes at 3,000 rpm at 4°C. Plasma was separated from blood cells and kept frozen at -20°C until assayed. *ANP *in plasma was determined by radioimmunoassay (RIA), as previously described [26]. ANP was extracted from plasma with C18 September-Pack (Water Associates, Milford, MA, USA) using ethanol, acetic acid, and water. For RIA, rabbit anti-ANP antibody was obtained from the Department of Clinical Chemistry, Bispebjerg Hospital, Denmark. The minimal detection concentration was 0.5 pmol l^-1^. The coefficients of variation were 12% (inter-assay) and 10% (intra-assay). *BNP *in plasma was determined by RIA as previously described [27]. Immunoreactive BNP was extracted from plasma with C18 September-Pack (Water Associates, Milford, MA, USA) eluted by 80% ethanol in a 4% acetic acid solution. A rabbit anti-BNP antibody without cross-reactivity with urodilatin or α-ANP was developed in our lab. The minimal detection concentration was 0.5 pmol l^-1^. The coefficients of variation were 11% (inter-assay) and 6% (intra-assay). *AVP *in plasma was measured by RIA using a modification of the method described previously [28]. AVP was extracted from plasma with C18 September-Pack (Water Associates, Milford, MA, USA). The antibody against AVP was a gift from Professor Jacques Dürr, Miami, FL, USA. The minimal detection concentration was 0.5 pmol l^-1^. The coefficients of variation were 13% (inter-assay) and 9% (intra-assay). *Ang II *in plasma was determined by RIA using a modification [29] of the method originally described [30]. Ang II was extracted from plasma with C18 September-Pack (Water Associates, Milford, MA, USA). The antibody against Ang II was obtained from the Department of Clinical Physiology, Glostrup Hospital, Denmark. The minimal detection concentration was 2 pmol l^-1^. The coefficients of variation were 12% (inter-assay) and 8% (intra-assay). *Aldo *was determined by RIA using a commercial kit (Diagnostic Systems Laboratories Inc, Webster, TX, USA). The minimal detection concentration was 22 pmol l^-1^. The coefficients of variation were 8.2% (inter-assay) and 3.9% (intra-assay). *PRC *was also determined by a commercial RIA (CIS Bio International, Gif-sur-Yvette Cedex, France). The minimal detection concentration was 0.5 pg ml^-1^. The coefficients of variation were 14.5% (inter-assay) and 4.5% (intra-assay).

Urine samples were centrifuged for 5 minutes at 3,000 rpm, and 125-1000 μl of the supernatant was freeze dried and kept frozen at -20°C for two to eight months until assayed. We have done pilot experiments to ensure that the concentration of the effect variables in the urine does not decrease over time. The experiments showed that the effect variables are stable at -20°C for two years. *U-AQP2 *was measured by a RIA as previously described [31]. The anti-AQP2 antibody for RIA was obtained from Søren Nielsen (The Water and Salt Research Center, Institute of Anatomy, Aarhus University, Denmark). The antibody was raised in rabbits against the 15 C-terminal amino-acids of human AQP2. The minimal detection concentration was 32 pg tube^-1^. The coefficients of variation were 11.7% (inter-assay) and 5.9% (intra-assay). *U-ENaC_β _*was measured by a newly developed RIA [7,32]. ENaC_β _was synthesized and purchased by Lofstrand Labs Limited (Gaithersburg, MD, USA). The β-ENaC antibody was raised against a synthetic peptide in rabbits. The lower detectable limit of the assay was 34 pg tube^-1^. The inter-assay variation was 12% at a mean level of 78 pg tube^-1^, and 10% at a mean level of 155 pg tube^-1^. The intra-assay variation was 6.4% and 9.0% at a mean level of 180 pg tube^-1 ^and 406 pg tube^-1^, respectively. *U-cAMP *was measured by RIA using a commercial kit (Biomedical Technologies Inc., Stoughton, MA, USA). The minimal detection concentration was 0.05 pmol l^-1^. The coefficients of variation were 8% (inter-assay) and 3% (intra-assay). *U-PGE_2 _*was measured by RIA using a commercial kit (Assay Designs, Inc., Ann Arbor, MI, USA). The minimal detection concentration was 8.26 pg ml^-1^. The coefficients of variation were 10.9% (inter-assay) and 6.3% (intra-assay).

*S-osm and u-osm *were measured by freezing-point depression (Advanced Model 3900 multisampling osmometer). *C_H2O _*was determined according to the formula: C_H2O _= V - C_osm_, where V is the urine output, and C_osm _is the osmolality clearance.

Urine was screened for blood, albumin, and glucose with standard urine test strips. Plasma and urinary concentrations of *sodium *and *creatinine *were determined at the Department of Clinical Biochemistry, Holstebro Hospital, Denmark using conventional methods. All clearances were standardized to a body surface area of 1.73 m^2^. *GFR *was measured using the constant infusion clearance technique with 51Cr-EDTA as reference.

*24-h ambulatory blood pressure *was measured with BOSO TM-2450 (Kiwex, Denmark).

*Clinic blood pressure *was measured with UA-743 digital blood pressure meter (A&D Company, Tokyo, Japan).

### Statistics

Statistical analyses were performed using SPSS version 15 (SPSS Inc., Chicago, IL, USA). Single baseline values were obtained by taking the weighted average of the measurements from the two baseline periods. The baseline values of the two groups were compared by Student's t-test. The baseline values during HS and LS intake were compared by paired samples t-tests. We used the "General Linear Model Repeated Measures" procedure in SPSS with time as within-subject factor and blood pressure as between-subject factor to compare the effect of blood pressure. The changes in response to the hypertonic saline infusion in each group were analyzed with the "General Linear Model Repeated Measures" procedure with time as within-subject factor and paired samples t-tests with Bonferroni correction as post hoc tests. P values < 0.05 were considered significant. Variables are normally distributed and presented as means with standard deviations (SD) or 95% confidence intervals, if not otherwise stated.

## Results

### Demographics

Twenty five patients with essential hypertension and 25 normotensive controls were enrolled in the study. Four patients were withdrawn from the study because they withdrew their consent to participation. Five normotensive controls were withdrawn from the study, two because of failure to obtain intravenous access, and three because of withdrawal of consent to participation. Table 1 shows the clinical and laboratory data of the 21 patients and 20 controls completing the study. The patients with essential hypertension had significantly higher systolic and diastolic blood pressure during both day and night compared with the controls. No significant difference in heart rate was found between patients and controls. Of the 21 patients, 16 were dippers (nocturnal BP fall ≥ 10%) and 5 were non-dippers (nocturnal BP fall < 10%). All the control subjects had a p-creatinine within the normal range (i.e. 50-90 μmol/l for women and 60-105 μmol/l for men).

**Table 1:** Clinical and laboratory characteristics of 21 patients with essential hypertension and 20 normotensive controls

	Patients	Controls	t-test
Men/women	14/7	12/8	

Age (years)	55 (9)	48 (9)	P = 0.102

BMI (kg m^-2^)	25.6 (2.5)	24.2 (2.7)	P = 0.160

p-sodium (mmol l^-1^)	139.4 (2.1)	139.6 (2.2)	P = 0.798

p-potassium (mmol l^-1^)	3.9 (0.4)	3.9 (0.3)	P = 0.992

p-creatinine (μmol l^-1^)	74.9 (9.4)	72.9 (10.9)	P = 0.532

24-h ambulatory			

SBP (mmHg)	144 (11)	119 (6)	P < 0.001

DBP (mmHg)	87 (7)	75 (4)	P < 0.001

HR (beats min^-1^)	71 (13)	68 (7)	P = 0.503

Daytime ambulatory			

SBP (mmHg)	148 (11)	124 (7)	P < 0.001

DBP (mmHg)	90 (8)	78 (4)	P < 0.001

HR (beats min^-1^)	74 (13)	71 (7)	P = 0.468

Nighttime ambulatory			

SBP (mmHg)	126 (12)	104 (12)	P < 0.001

DBP (mmHg)	76 (9)	63 (4)	P < 0.001

HR	59 (10)	56 (9)	P = 0.487

Values are means (SD). Student's t-test.

### 24-h urine collection

Table 2 shows the results of the 24-h urine collection in the patients with essential hypertension and the normotensive controls during HS and LS intake. U-AQP2_CR _and u-ENaC_β-CR _were the same in patients and controls in the 24-h urine. U-AQP2_CR _was significantly lower during LS intake than HS intake in both patients and controls, whereas u-ENaC_β-CR _was the same during LS and HS. The only significant difference between patients and controls was a significantly lower u-osm in the patients during LS intake. U_Na _was significantly lower in both patients and controls during LS intake compared to during HS intake, indicating that both groups had kept the supplied diets.

**Table 2:** 24-hours urine collection

			t-test
***U_Na _(μmol min^-1^)***			

HS	Patients	203 (179; 227)§	P = 0.215
		
	Controls	226 (195; 257)§	

LS	Patients	42 (31; 53)	P = 0.494
		
	Controls	47 (35; 59)	

***u-EnaC_β-CR _(pg μmol^-1^)***			

HS	Patients	14.33 (12.30; 16.37)	P = 0.980
		
	Controls	14.37 (12.34; 16.40)	

LS	Patients	12.54 (11.35; 13.74)	P = 0.084
		
	Controls	14.19 (13.00; 15.38)	

***V (ml min^-1^)***			

HS	Patients	2702 (2331; 3074)	P = 0.628
		
	Controls	2828 (2442; 3213)	

LS	Patients	2991 (2721; 3260)	P = 0.207
		
	Controls	2715 (2353; 3078)	

***u-AQP2_CR _(ng mmol^-1^)***			

HS	Patients	143 (117; 168)§	P = 0.781
		
	Controls	147 (129; 165)§	

LS	Patients	101 (89; 114)	P = 0.192
		
	Controls	112 (101; 122)	

***u-osm (mOsm kg^-1^)***			

HS	Patients	405 (363; 447)§	P = 0.409
		
	Controls	436 (372; 499)§	

LS	Patients	221 (204; 238)	P = 0.026
		
	Controls	306 (231; 381)	

Values are means with 95% confidence intervals in brackets. U_Na_, urinary sodium excretion rate; u-ENaC_β__-CR_, urinary ENaC_β _excretion corrected for creatinine; V, urinary flow; u-AQP2_CR_, urinary AQP2 excretion corrected for creatinine; u-osm, urine osmolality; t-test: patients compared with controls, Student's t-test. § P < 0.001, HS vs. LS intake, paired samples t-test.

### Hypertonic saline infusion

The effect variables are shown in Table 3 (U_Na_, FE_Na _and u-ENaC_β-CR_), Table 4 (V, C_H2O_, u-AQP2_CR_, u-osm, u-c-AMP, u-PGE_2_, and s-osm), Table 5 (PRC, AngII, Aldo, ANP, BNP and AVP), and Table 6 (GFR, SBP, DBP and HR) before (baseline), and after the hypertonic saline infusions in the patients with essential hypertension and the normotensive controls during HS and LS intake, respectively. The relative changes in the effect variables are shown in Tables 7 and 8.

**Table 3:** Effects of hypertonic saline infusion (3%, 7 ml kg^-1^) on U_Na_, FE_Na_, and u-ENaC _β-CR _in 21 patients with essential hypertension and 20 normotensive controls during high and low sodium intake

		Baseline	60 Min	120 Min	180 Min	P_GLM RM_
***U_Na _(μmol min^-1^)***						

HS	Patients	343 (300; 386)§	615 (499; 731)†	700 (581; 819)†	541 (471; 611)†	P = 0.110
		
	Controls	323 (285; 361)§	497 (321; 672)†	499 (357; 642)†	460 (366; 554)†	

LS	Patients	91 (57; 125)	254 (168; 340)†	275 (192; 357)†	277 (211; 343)†	P = 0.164
		
	Controls	57 (40; 74)	175 (62; 288)†	191 (99; 282)†	181 (123; 239)†	

***FE_Na _(%)***						

HS	Patients	2.66 (2.34; 2.98)§	5.10 (4.20; 6.00)†	5.35 (4.46; 6.24)†	4.57 (3.96; 5.19)†	P = 0.030
		
	Controls	2.51 (2.10; 2.92)§	3.73 (2.61; 4.86)†	3.72 (2.76; 4.68)†	3.49 (2.85; 4.14)†	

LS	Patients	0.75 (0.49; 1.01)	2.24 (1.55; 2.93)†	2.29 (1.69; 2.90)†	2.28 (1.84; 2.72)†	P = 0.059
		
	Controls	0.49 (0.34; 0.65)	1.34 (0.67; 2.01)†	1.52 (0.90; 2.15)†	1.44 (1.06; 1.82)†	

***u-EnaC_β-CR _(pg μmol^-1^)***						

HS	Patients	11.4 (10.2; 12.7)§	10.4 (9.4; 11.3)	10.8 (9.6; 12.1)	11.1 (9.6; 12.6)	P = 0.796
		
	Controls	11.2 (9.9; 12.5)	10.8 (9.7; 12.0)	12.2 (10.7; 13.8)†	11.0 (9.4; 12.6)	

LS	Patients	10.4 (9.4; 11.3)	10.7 (9.3; 12.2)	11.5 (10.4; 12.6)†	10.9 (10.0; 11.9)	P = 0.716
		
	Controls	11.5 (9.8; 13.2)	11.2 (10.0; 12.4)	12.4 (11.3; 13.6)†	11.9 (10.2; 13.5)	

Values are means with 95% confidence intervals in brackets. U_Na_, urinary sodium excretion rate; FE_Na_, fractional sodium excretion; u-ENaC_β-CR_, urinary ENaC_β _excretion corrected for creatinine. P_GLM RM_: patients compared with controls, GLM repeated measures with time as within-subject factor and blood pressure as between-subject factor. * P < 0.05, baseline patients vs. controls, Student's t-test. § P < 0.05, baseline HS vs. LS, paired samples t-test. † P < 0.05, compared with baseline, paired samples t-test

**Table 4:** Effects of hypertonic saline infusion (3%, 7 ml kg^-1^) on V, C_H2O_, u-AQP2_CR_, u-osm, u-cAMP, u-PGE_2_, and s-osm in 21 patients with essential hypertension and 20 normotensive controls during high and low sodium intake

		Baseline	60 Min	120 Min	180 Min	P_GLM RM_
***V (ml min^-1^)***						

HS	Patients	8.7 (7.7; 9.6)§	5.0 (4.0; 6.0)†	4.1 (3.3; 4.8)†	3.3 (2.8; 3.8)†	P = 0.076
		
	Controls	8.1 (7.4; 8.7)§	4.1 (3.1; 5.0)†	2.9 (2.2; 3.6)†	3.3 (2.6; 4.0)†	

LS	Patients	7.4 (6.6; 8.2)	4.0 (2.6; 5.4)†	2.8 (1.6; 4.1)†	2.8 (2.1; 3.4)†	P = 0.078
		
	Controls	6.6 (5.9; 7.2)	2.7 (1.9; 3.5)†	1.8 (1.1; 2.4)†	2.3 (1.7; 2.9)†	

***C_H2O _(ml min^-1^)***						

HS	Patients	4.1 (3.1; 5.0)	-0.6 (-1.2; 0.0)†	-2.3 (-2.8; -1.9)†	-1.9 (-2.3; -1.5)†	P = 0.495
		
	Controls	3.5 (2.7; 4.3)	-1.0 (-1.4; -0.5)†	-2.2 (-2.7; -1.7)†	-1.6 (-2.2; -1.1)†	

LS	Patients	4.6 (4.0; 5.3)	0.9 (-0.2; 1.9)†	-0.6 (-1.6; 0.5)†	-0.7 (-1.4; 0.0)†	P = 0.216
		
	Controls	4.0 (3.3; 4.7)	0.1 (-0.2; 0.4)†	-0.9 (-1.3; -0.5)†	-0.6 (-1.0; -0.1)†	

***u-AQP2_CR _(ng mmol^-1^)***						

HS	Patients	154 (137; 172)§	206 (179; 233)†	220 (196; 243)†	201 (179; 222)†	P = 0.055
		
	Controls	156 (140; 172)§	162 (146; 177)	186 (155; 217)†	175 (151; 199)†	

LS	Patients	109 (87; 130)	136 (120; 153)†	140 (125; 155)†	135 (125; 145)†	P = 0.589
		
	Controls	118 (107; 130)	120 (94; 147)	129 (105; 153)	122 (105; 139)	

***u-osm (mOsm kg^-1^)***						

HS	Patients	169 (130; 209)§	345 (311; 379)†	484 (445; 522)†	479 (431; 526)†	P = 0.237
		
	Controls	172 (149; 194)§	371 (329; 413)†	542 (485; 599)†	486 (411; 562)†	

LS	Patients	111 (98; 124)	263 (221; 305)†	433 (368; 497)†	405 (343; 467)†	P = 0.231
		
	Controls	121 (101; 141)	271 (240; 303)†	497 (413; 581)†	438 (348; 529)†	

***u-cAMP (nmol min^-1^)***						

HS	Patients	7.10 (6.04; 8.16)§	6.26 (5.58; 6.94)	5.87 (5.16; 6.58)†	5.62 (4.91; 6.33)†	P = 0.388
		
	Controls	5.87 (4.95; 6.79)	6.02 (5.16; 6.87)	5.41 (4.73; 6.09)	5.34 (4.67; 6.02)	

LS	Patients	6.34 (5.39; 7.30)	5.91 (5.24; 6.57)	5.19 (4.51; 5.87)†	5.25 (4.55; 5.95)†	P = 0.240
		
	Controls	5.64 (4.58; 6.70)	4.91 (3.87; 5.95)†	4.30 (3.61; 4.99)†	4.67 (3.93; 5.40)†	

***u-PGE_2 _(μmol min^-1^)***						

HS	Patients	1095 (862; 1329)§	980 (383; 1577)	754 (590; 917)†	499 (418; 581)†	P = 0.772
		
	Controls	1180 (843; 1516)§	868 (681; 1055)†	752 (559; 945)†	653 (454; 851)†	

LS	Patients	2294 (1103; 3485)	1276 (864; 1687)†	648 (490; 806)†	679 (549; 809)†	P = 0.505
		
	Controls	1487 (1049; 1926)	1072 (850; 1295)†	736 (536; 935)†	810 (602; 1018)†	

***s-osm (mOsm kg^-1^)***						

HS	Patients	290 (289; 292)§	297 (296; 299)†	294 (292; 295)†	290 (289; 292)	P = 0.263
		
	Controls	290 (289; 291)§	296 (295; 297)†	292 (291; 293)†	290 (288; 291)	

LS	Patients	286 (283; 289)	294 (292; 296)†	292 (290; 294)†	289 (287; 291)†	P = 0.734
		
	Controls	286 (285; 288)	293 (292; 295)†	291 (289; 292)†	288 (287; 290)†	

Values are means with 95% confidence intervals in brackets. V, urinary flow; C_H2O_, free water clearance; u-AQP2_CR_, urinary AQP2 excretion corrected for creatinine; u-osm, urine osmolality; u-cAMP, urinary cAMP excretion; and u-PGE_2_, urinary prostaglandin E2 excretion; and s-osm, serum osmolality. P_GLM RM_: patients compared with controls, GLM repeated measures with time as within-subject factor and blood pressure as between-subject factor. * P < 0.05, baseline patients vs. controls, Student's t-test. § P < 0.05, baseline HS vs. LS, paired samples t-test. † P < 0.05, compared with baseline, paired samples t-test

**Table 5:** Effects of hypertonic saline infusion (3%, 7 ml kg^-1^) on PRC, Ang II, Aldo, ANP, BNP, and AVP in 21 patients with essential hypertension and 20 normotensive controls during high and low sodium intake

		Baseline	60 Min	120 Min	180 Min	P_GLM RM_
***PRC (mU l^-1^)***						

HS	Patients	4.8 (2.9; 6.7)§	3.8 (2.5; 5.1)†	3.5 (2.0; 5.1)	4.2 (2.1; 6.3)	P = 0.311
		
	Controls	6.5 (3.1; 9.8)§	4.4 (3.0; 5.7)	4.8 (2.4; 7.2)	5.5 (2.5; 8.6)	

LS	Patients	13.6 (8.5; 18.8)	8.9 (5.8; 12.0)	6.6 (4.7; 8.4)†	8.2 (4.8; 11.6)	P = 0.145
		
	Controls	19.8 (13.5; 26.0)	11.1 (8.2; 14.0)†	11.5 (4.7; 18.3)	11.2 (4.5; 17.8)	

***AngII (pmol l^-1^)***						

HS	Patients	4.0 (3.2; 4.7)*§	3.4 (2.3; 4.5)	3.5 (2.3; 4.7)	3.4 (2.4; 4.3)	P = 0.079
		
	Controls	6.1 (4.4; 7.8)§	4.6 (2.8; 6.4)	4.4 (3.4; 5.4)	4.2 (3.1; 5.2)	

LS	Patients	11.7 (7.3; 16.0)	6.4 (4.5; 8.3)†	5.6 (4.5; 6.7)†	5.7 (4.1; 7.3)†	P = 0.080
		
	Controls	14.4 (11.1; 17.6)	9.3 (5.6; 12.9)†	8.8 (6.2; 11.3)†	7.7 (5.8; 9.6)†	

***Aldo (pmol l^-1^)***						

HS	Patients	227 (192; 261)§	173 (147; 199)†	168 (144; 192)†	175 (152; 199)†	P = 0.063
		
	Controls	188 (154; 221)§	145 (123; 166)†	137 (120; 155)†	151 (131; 170)	

LS	Patients	352 (275; 428)	237 (196; 279)†	214 (185; 244)†	231 (202; 260)†	P = 0.973
		
	Controls	365 (296; 434)	238 (193; 284)†	211 (178; 244)†	216 (188; 245)†	

***ANP (pmol l^-1^)***						

HS	Patients	11.7 (9.6; 13.9)§	16.3 (12.5; 20.1)	14.4 (11.5; 17.4)†	12.8 (10.2; 15.4)†	P = 0.415
		
	Controls	11.1 (9.5; 12.7)§	14.4 (12.1; 16.7)	12.9 (11.1; 14.8)†	11.6 (9.9; 13.3)†	

LS	Patients	7.4 (5.9; 8.8)	11.1 (8.6; 13.7)†	10.1 (7.8; 12.4)†	9.1 (7.2; 11.0)†	P = 0.707
		
	Controls	7.3 (5.8; 8.8)	10.4 (8.7; 12.0)†	9.5 (7.9; 11.0)†	8.9 (7.3; 10.4)†	

***BNP (pmol l^-1^)***						

HS	Patients	2.2 (0.8; 3.7)§	2.6 (0.9; 4.2)	2.4 (1.1; 3.8)†	2.5 (1.1; 3.8)	P = 0.938
		
	Controls	2.2 (0.5; 3.9)§	2.5 (0.6; 4.4)	2.6 (0.5; 4.7)	2.7 (0.5; 4.9)	

LS	Patients	0.9 (0.6; 1.2)	1.0 (0.6; 1.4)	1.1 (0.7; 1.4)	1.1 (0.7; 1.5)	P = 0.941
		
	Controls	1.0 (0.4; 1.5)	1.0 (0.5; 1.6)	1.1 (0.5; 1.7)	1.1 (0.5; 1.7)†	

***AVP (pg ml^-1^)***						

HS	Patients	0.69 (0.60; 0.77)	1.08 (0.91; 1.24)†	0.87 (0.75; 0.99)†	0.70 (0.60; 0.79)	P = 0.520
		
	Controls	0.66 (0.57; 0.75)	0.97 (0.82; 1.12)†	0.79 (0.66; 0.92)†	0.72 (0.60; 0.83)	

LS	Patients	0.65 (0.57; 0.73)	1.04 (0.88; 1.19)†	0.86 (0.75; 0.96)†	0.73 (0.64; 0.83)	P = 0.535
		
	Controls	0.66 (0.57; 0.75)	0.93 (0.75; 1.11)†	0.78 (0.66; 0.90)†	0.73 (0.56; 0.89)	

Values are means with 95% confidence intervals in brackets. PRC, plasma renin concentration; Ang II, angiotensin II, Aldo, aldosterone; ANP, atrial natriuretic peptide; and BNP, brain natriuretic peptide before (baseline) and 60, 120, and 180 minutes after a hypertonic saline infusion. P_GLM RM_: patients compared with controls, GLM repeated measures with time as within-subject factor and blood pressure as between-subject factor. * P < 0.05, baseline patients vs. controls, Student's t-test. § P < 0.05, baseline HS vs. LS, paired samples t-test. † P < 0.05, compared with baseline, paired samples t-test

**Table 6:** Effects of hypertonic saline infusion (3%, 7 ml kg^-1^) on GFR, blood pressure, and heart rate in 21 patients with essential hypertension and 20 normotensive controls during high and low sodium intake

		Baseline	60 Min	120 Min	180 Min	P_GLM RM_
***Glomerular filtration rate (ml min^-1^)***						

HS	Patients	93 (87; 100)§	85 (78; 91)	93 (85; 101)	86 (79; 93)	P = 0.321
		
	Controls	96 (88; 104)§	90 (82; 98)	94 (86; 102)	93 (88; 99)	

LS	Patients	86 (81; 90)	78 (73; 84)†	84 (75; 93)	86 (77; 94)	P = 0.458
		
	Controls	89 (80; 98)	81 (72; 90)	83 (72; 94)	91 (81; 101)	

***Systolic blood pressure (mmHg)***						

HS	Patients	142 (134; 150)*§	148 (141; 155)†	145 (138; 151)	149 (141; 157)†	P < 0.001
		
	Controls	116 (110; 121)	119 (113; 125)	117 (111; 123)	119 (112; 125)	

LS	Patients	135 (129; 141)*	139 (133; 145)	138 (132; 145)	136 (130; 142)	P < 0.001
		
	Controls	114 (110; 118)	116 (110; 121)	115 (110; 120)	118 (112; 124)	

***Diastolic blood pressure (mmHg)***						

HS	Patients	86 (81; 91)*	87 (81; 92)	87 (82; 92)	89 (83; 94)	P < 0.001
		
	Controls	70 (66; 73)	70 (66; 74)	70 (66; 74)	72 (69; 76)†	

LS	Patients	84 (80; 88)*	83 (78; 88)	84 (79; 89)	85 (80; 89)	P < 0.001
		
	Controls	69 (66; 72)	68 (65; 71)	67 (64; 71)	70 (67; 73)	

***Heart rate (beats min^-1^)***						

HS	Patients	56 (52; 60)	57 (53; 62)	58 (53; 63)	57 (54; 61)	P = 0.204
		
	Controls	52 (47; 57)	53 (49; 57)	54 (50; 59)†	54 (50; 59)	

LS	Patients	57 (53; 61)	59 (54; 63)	59 (55; 63)	59 (55; 62)	P = 0.261
		
	Controls	53 (49; 57)	56 (51; 60)†	57 (52; 61)†	56 (52; 60)†	

Values are means (95% confidence intervals). P_GLM RM_: patients compared with controls, GLM repeated measures with time as within-subject factor and blood pressure as between-subject factor. * P < 0.05, baseline patients vs. controls, Student's t-test. § P < 0.05, baseline HS vs. LS, paired samples t-test. † P < 0.05, compared with baseline, paired samples t-test

**Table 7:** Relative changes from baseline in U_Na_, FE_Na_, and u-ENaC_β-CR _in response to a hypertonic saline infusion (3%, 7 ml kg^-1^) in 21 patients with essential hypertension and 20 normotensive controls during HS and LS intake

		60 Min	120 Min	180 Min	P_GLM RM_
***ΔU_Na _(%)***					

HS	Patients	87.4 (49.5; 125.4)§	112.4 (72.0; 152.8)*§	64.6 (41.4; 87.7)§	P = 0.122
		
	Controls	48.2 (7.8; 88.6)§	54.6 (18.4; 90.8)§	44.0 (20.2; 67.9)§	

LS	Patients	388.9 (108.6; 669.2)	475.2 (133.8; 816.6)	513.7 (145.9; 881.5)	P = 0.363
		
	Controls	241.3 (92.9; 389.7)	306.8 (109.3; 504.3)	379.0 (168.4; 589.5)	

***ΔFE_Na _(%)***					

HS	Patients	96.6 (63.2; 130.1)§	107.4 (72.8; 142.1)*§	75.8 (56.2; 95.4)§	P = 0.047
		
	Controls	50.6 (14.0; 87.1)§	54.1 (21.0; 87.3)§	46.2 (20.6; 71.7)§	

LS	Patients	379.9 (145.4; 614.4)	442.9 (142.6; 743.2)	486.0 (166.5; 805.5)	P = 0.323
		
	Controls	238.7 (106.8; 370.6)	286.5 (120.0; 453.0)	365.3 (157.5; 573.1)	

***Δu-ENaC_β-CR _(%)***					

HS	Patients	-5.2 (-15.7; 5.2)	-4.1 (-12.7; 3.8)	-3.5 (-12.0; 5.1)	P = 0.311
		
	Controls	-1.7 (-9.6; 6.1)	8.3 (-7.6; 23.9)	-1.8 (-10.8; 7.2)	

LS	Patients	5.2 (-5.7; 16.2)	13.9 (5.0; 22.7)	7.9 (-0.2; 16.0)	P = 0.525
		
	Controls	-1.5 (-16.3; 13.2)	8.1 (-12.3; 28.5)	7.0 (-7.3; 21.3)	

U_Na_, urinary sodium excretion rate; FE_Na_, fractional sodium excretion; u-ENaC_β-CR_, urinary ENaC_β _excretion corrected for creatinine. Values are means (95% confidence intervals). P_GLM RM_: HS compared with LS, GLM Repeated Measures with time as within-subject factor and blood pressure as between-subject factor. * P < 0.05, patients vs. controls, Students t-test. § P < 0.05, HS compared with LS, paired samples t-test

**Table 8:** Relative changes from baseline in V, C_H2O_, u-AQP2_CR_, u-osm, u-cAMP, u-PGE_2_, and s-osm in response to a hypertonic saline infusion (3%, 7 ml kg^-1^) in 21 patients with essential hypertension and 20 normotensive controls during HS and LS intake

		60 Min	120 Min	180 Min	P_GLM RM_
***ΔV (%)***					

HS	Patients	-37.9 (-52.8; -23.0)	-48.8 (-61.7; -35.9)	-57.9 (-67.4; -48.3)	P = 0.212
		
	Controls	-50.1 (-60.4; -39.8)	-63.5 (-71.3; -55.7)§	-58.9 (-67.3; -50.4)	

LS	Patients	-43.6 (-62.7; -24.6)	-60.5 (-77.9; -43.0)	-59.9 (-70.2; -49.6)	P = 0.487
		
	Controls	-55.9 (-69.2; -42.6)	-70.6 (-82.0; -59.3)	-62.4 (-74.4; -50.4)	

***ΔC_H2O _(%)***					

HS	Patients	-111.0 (-130.7; -91.3)§	-152.7 (-174.3; -131.1)§	-142.5 (-158.4; -126.6)§	P = 0.715
		
	Controls	-130.3 (-161.7; -100.0)§	-164.7 (-227.2; -102.1)	-141.5 (-238.4; -44.5)	

LS	Patients	-78.5 (-102.0; -55.0)	-112.1 (-135.8; -88.3)	-113.4 (-128.0; -98.8)	P = 0.519
		
	Controls	-90.9 (-103.7; -78.1)	-121.8 (-131.3; -112.2)	-112.5 (-125.5; -99.5)	

***Δu-AQP2_CR _(%)***					

HS	Patients	35.0 (23.2; 46.7)*	46.2 (30.5; 61.9)*	31.9 (22.1; 41.7)*	P = 0.005
		
	Controls	6.1 (-4.5; 16.8)	22.2 (7.1; 37.4)§	12.6 (1.5; 23.8)	

LS	Patients	45.9 (10.0; 81.9)*	50.1 (17.0; 83.1)*	41.2 (15.8; 66.6)*	P = 0.019
		
	Controls	-1.1 (-13.5; 11.3)	6.7 (-6.2; 19.6)	3.0 (-5.5; 11.6)	

***Δu-osm (%)***					

HS	Patients	134.7 (88.4; 181.0)	232.7 (168.4; 297.1)§	228.6 (164.1; 293.1)§	P = 0.757
		
	Controls	125.5 (97.1; 153.9)	235.5 (179.0; 292.0)§	197.6 (142.1; 253.1)§	

LS	Patients	146.6 (103.6; 189.7)	315.3 (232.1; 398.5)	287.6 (211.8; 363.4)	P = 0.661
		
	Controls	141.6 (103.2; 180.0)	362.0 (252.9; 471.1)	297.4 (206.2; 388.6)	

***Δu-cAMP (%)***					

HS	Patients	-6.4 (-18.7; 5.9)	-12.9 (-21.6; -4.1)	-16.4 (-25.6; -7.3)	P = 0.178
		
	Controls	5.9 (-7.2; 19.0)	-10.7 (-23.8; 2.5)§	-6.4 (-14.3; 1.4)	

LS	Patients	-3.0 (-12.3; 6.3)	-12.5 (-25.6; 0.6)	-13.6 (-22.8; -4.4)	P = 0.256
		
	Controls	-13.0 (-27.9; 2.0)	-28.6 (-44.7; -12.6)	-13.9 (-26.2; -1.7)	

***Δu-PGE_2 _(%)***					

HS	Patients	11.1 (-70.9; 93.1)	-20.8 (-39.4; -2.1)	-46.3 (-56.4; -36.1)	P = 0.410
		
	Controls	-22.0 (-33.2; -10.8)	-34.6 (-44.3; -24.9)	-43.4 (-50.6; -36.3)	

LS	Patients	-9.4 (-38.2; 19.3)	-38.0 (-63.4; -12.6)	-40.9 (-59.3; -22.4)	P = 0.689
		
	Controls	-16.1 (-34.4; 2.1)	-45.0 (-56.3; -33.7)	-41.6 (-51.1; -32.2)	

***Δs-osm (%)***					

HS	Patients	2.4 (2.0; 2.9)	1.2 (0.8; 1.6)§	0.0 (-0.4; 0.3)§	P = 0.671
		
	Controls	2.2 (1.9; 2.5)	0.9 (0.6; 1.2)§	0.1 (-0.3; 0.6)§	

LS	Patients	2.7 (2.3; 3.1)	2.0 (1.5; 2.5)	0.9 (0.4; 1.4)	P = 0.215
		
	Controls	2.5 (2.2; 2.8)	1.6 (1.2; 1.9)	0.7 (0.4; 1.0)	

V, urinary flow; C_H2O_, free water clearance; u-AQP2_CR_, urinary AQP2 excretion corrected for creatinine; u-osm, urinary osmolality; u-c-AMP, urinary cyclic-AMP excretion; u-PGE_2_, urinary prostaglandin E2 excretion; s-osm, serum osmolality. Values are means (95% confidence intervals). P_GLM RM_: HS compared with LS, GLM Repeated Measures with time as within-subject factor and blood pressure as between-subject factor. * P < 0.05, patients vs. controls, Students t-test. § P < 0.05, HS compared with LS, paired samples t-test

#### Baseline results

We found no difference in the above mentioned effect parameters between patients and controls at baseline, except lower Ang II in patients compared with controls during HS intake (Table 5) and as expected higher systolic and diastolic blood pressure during HS intake compared with LS intake in patients, but not in controls. LS intake resulted in extracellular volume depletion in both groups. Thus during LS intake, we found significantly lower U_Na _and FE_Na _(Table 3), significantly lower V, u-osm, and s-osm (Table 4), significantly lower ANP and BNP, and significantly higher PRC, ANG II and Aldo (Table 5), and significantly lower GFR (Table 6) at baseline in both groups. Moreover, u-AQP2_CR _was significantly lower and u-PGE_2 _significantly higher during LS intake compared with during HS intake in both patients and controls (Table 4).

U-ENaC_β-CR _and u-cAMP were significantly lower during LS intake compared with HS intake in the patients at baseline, but the differences were very small. U-ENaC_β-CR _and u-cAMP were the same in the control group at baseline (Table 3 and 4).

Systolic blood pressure was significantly lower during LS intake than HS intake at baseline in the patient group, but not in the control group (Table 6).

#### Sodium excretion and u-ENaC_β-CR_

The hypertonic saline infusion was accompanied by significant increases in U_Na _and FE_Na _in both patients and controls during both HS and LS intake. The increases lasted throughout the experiment (Table 3). During HS intake, the relative increases in both U_Na _and FE_Na _were significantly higher in the patients with essential hypertension than in the controls 120 minutes after infusion start (Table 7). The relative increases in U_Na _and FE_Na _were significantly higher during LS intake compared with HS intake throughout the experiment in both groups (Table 7).

A significant increase was seen in u-ENaC_β-CR _in patients with essential hypertension during LS intake and in the normotensive controls during both HS and LS intake 120 minutes after the saline infusion start (Table 3). There was no difference between patients and controls with regard to the relative increases (Table 7).

#### Water excretion, u-AQP2_CR_, u-osm, u-cAMP, u-PGE_2_, and s-osm

In both patients and controls, the hypertonic saline infusion induced a significant and sustained decrease in V (Table 4). The relative decreases in V did not differ between patients and controls (Table 8).

C_H2O _decreased significantly after the hypertonic saline infusion in both groups and during both diets with a maximum after 120 minutes (Table 4). C_H2O _changed from positive values at baseline to negative values after infusion, indicating a change from water excretion to water reabsorption. The relative decreases in C_H2O _did not differ between patients and controls. In the patients with essential hypertension the relative changes were significantly higher during HS intake than during LS intake throughout the experiment. In the normotensive controls, this was only the case 60 minutes after infusion start (Table 8).

U-AQP2_CR _increased significantly in response to the hypertonic saline infusion in both patients and controls during both diets (Table 4). The increase reached maximum 120 minutes after the infusion start. The relative increases were significantly higher in the patients than in the controls throughout the experiment (HS: 35 versus 6%; LS: 46 versus -1%) (Table 8).

U-osm increased significantly in response to the hypertonic saline infusion in both patients and controls during both diets (Table 4). The increases lasted throughout the experiment with a maximum 120 minutes after infusion start. The relative increases did not differ between patients and controls, but they were significantly higher during LS intake in both groups (Table 8).

U-cAMP decreased in response to the saline infusion in both patients and controls (Table 4). In the control subjects the decrease was not significant during HS intake, but there was no difference in the relative decrease between patients and controls during any of the diets (Table 8).

U-PGE_2 _decreased in response to the saline infusion in both patients and controls during both diets (Table 4) with a nadir 180 minutes after infusion start during HS intake and one hour earlier during LS intake. The relative decreases in u-PGE_2 _were not significantly different between patients and controls or between the two diets (Table 8).

S-osm increased significantly in both patients and controls during both diets with a maximum 60 minutes after infusion start. The maximal increase did not differ between patients and controls or between HS and LS intake (Table 8), but only during HS intake baseline levels were reached 180 minutes after infusion start.

#### Vasoactive hormones

In the patients, PRC decreased 60 minutes after infusion start during HS intake and one hour later during LS intake. PRC did also decrease in the normotensive controls, but the changes from baseline were only significant during LS intake (Table 5). The relative decreases in PRC did not differ between patients and controls or between HS and LS intake (Table 9).

**Table 9:** Relative changes from baseline in PRC, Ang II, Aldo, ANP, BNP, and AVP in response to a hypertonic saline infusion (3%, 7 ml kg^-1^) in 21 patients with essential hypertension and 20 normotensive controls during HS and LS intake

		60 Min	120 Min	180 Min	P_GLM RM_
***ΔPRC (%)***					

HS	Patients	-16.1 (-24.5; -7.7)	-16.3 (-37.4; 4.8)	-14.9 (-25.9; -3.9)	P = 0.487
		
	Controls	-9.2 (-29.4; 11.0)	-5.1 (-39.8; 29.5)	-7.4 (-40.4; 25.7)	

LS	Patients	-21.4 (-35.9; -6.8)	-35.2 (-53.7; -16.6)	-29.3 (-50.6; -8.0)	P = 0.526
		
	Controls	-30.2 (-49.4; -10.9)	-36.1 (-55.2; -16.9)	-39.0 (-57.8; -20.3)	

***ΔAngII (%)***					

HS	Patients	-16.4 (-30.3; -2.6)	-12.0 (-30.4; 6.4)§	-15.4 (-27.3; -3.5)§	P = 0.366
		
	Controls	-25.0 (-37.2; -12.8)	-14.3 (-33.1; 4.4)	-26.2 (-36.6; -15.8)§	

LS	Patients	-33.5 (-48.0; -18.9)	-34.7 (-50.1; -19.3)	-38.5 (-50.7; -26.2)	P = 0.845
		
	Controls	-36.8 (-48.1; -25.4)	-32.3 (-56.4; -8.2)	-45.1 (-52.3; -37.8)	

***ΔAldo (%)***					

HS	Patients	-21.7 (-28.7; -14.8)§	-24.3 (-30.6; -18.0)§	-20.1 (-26.5; -13.7)	P = 0.654
		
	Controls	-21.5 (-27.0; -15.9)§	-23.7 (-31.8; -15.6)§	-14.9 (-25.9; -4.0)§	

LS	Patients	-30.2 (-35.1; -25.4)	-34.8 (-40.8; -28.8)	-27.5 (-36.3; -18.7)	P = 0.128
		
	Controls	-33.5 (-38.0; -28.9)	-39.2 (-45.5; -32.9)	-36.8 (-43.9; -29.6)	

***ΔANP (%)***					

HS	Patients	38.6 (24.3; 53.0)	25.6 (13.2; 38.0)	10.9 (-1.0; 22.7)§	P = 0.319
		
	Controls	30.6 (16.3; 45.0)§	17.8 (8.3; 27.4)§	6.2 (-3.6; 16.1)§	

LS	Patients	60.3 (30.9; 89.7)	48.3 (18.5; 78.1)	28.2 (11.3; 45.1)	P = 0.481
		
	Controls	48.9 (33.4; 64.4)	35.0 (22.6; 47.4)	25.8 (15.5; 36.1)	

***ΔBNP (%)***					

HS	Patients	34.6 (-13.9; 83.1)	15.6 (9.4; 21.8)	22.0 (12.3; 31.8)	P = 0.903
		
	Controls	15.7 (0.8; 30.6)	22.0 (6.6; 37.4)	26.3 (7.1; 45.6)	

LS	Patients	15.7 (0.8; 30.6)	22.0 (6.6; 37.4)	26.3 (7.1; 45.6)	P = 0.305
		
	Controls	7.8 (0.9; 14.6)	14.9 (4.3; 25.6)	15.2 (5.1; 25.3)	

***ΔAVP (%)***					

HS	Patients	57.5 (40.9; 74.1)	28.3 (15.9; 40.6)	3.2 (-5.7; 12.1)	P = 0.666
		
	Controls	50.9 (31.8; 69.9)	19.0 (6.7; 31.2)	8.4 (-0.7; 17.5)	

LS	Patients	64.4 (38.6; 90.2)	36.1 (20.5; 51.6)	15.3 (4.5; 26.2)	P = 0.110
		
	Controls	41.5 (23.0; 59.9)	20.6 (8.1; 33.1)	9.7 (-4.9; 24.4)	

PRC, plasma renin concentration; Ang II, angiotensin II, Aldo, aldosterone; ANP, atrial natriuretic peptide; BNP, brain natriuretic peptide; AVP, vasopressin. Values are means (95% confidence intervals). P_GLM RM_: HS compared with LS, GLM Repeated Measures with time as within-subject factor and blood pressure as between-subject factor. * P < 0.05, patients vs. controls, Students t-test. § P < 0.05, HS compared with LS, paired samples t-test

In both patients and controls, Ang II decreased significantly in response to the hypertonic saline infusion during LS intake, but not during HS intake (Table 5). Accordingly, the relative decreases in Ang II were significantly larger during LS intake in both groups (Table 9).

Aldo decreased significantly in both groups during both diets. The decrease had a maximum 120 minutes after the saline infusion start and lasted throughout the study day (Table 5). The relative decreases were not different between patients and controls, but were significantly higher during LS intake in both groups (Table 9).

The hypertonic saline infusion induced a significant and sustained increase in ANP in both groups on both study days (Table 5). The relative increases in ANP were higher during LS intake than during HS intake (Table 9).

In the patients, BNP increased significantly 120 minutes after infusion start during HS intake, while BNP increased significantly 180 minutes after infusion start during LS intake in the controls. The relative increases did not differ between patients and controls or during HS and LS intake (Table 9).

AVP increased significantly after the saline infusion in both patients and controls during both diets (Table 5). The increases followed the same pattern in patients and controls with a maximum after 60 minutes (Table 5). The relative changes in AVP were not significantly different between patient and controls or between HS and LS intake throughout the study day (Table 9).

#### Blood pressure, heart rate, body weight, p-Na, and p-albumin

The systolic blood pressure increased significantly in response to the hypertonic saline during HS intake in the patients, but not in the controls (Table 6).

The hypertonic saline did not affect the diastolic blood pressure of the patients (Table 6). In the control group a significant increase in the diastolic blood pressure was seen 180 minutes after the saline infusion during HS intake (Table 6).

The heart rate of the patients did not increase significantly in response to the hypertonic saline infusion, while the heart rate of the controls increased significantly during both diets (Table 6). The relative increases in heart rate did not differ between patients and controls.

The body weight of the patients remained constant throughout the study day during HS intake (80.4 kg (SD 12.5)), while it increased from 79.3 kg (SD 12.3) to 79.9 kg (SD 12.3) during LS intake. The increase was statistically significant (0.6 kg (SD 0.5), P < 0.001). The body weight of the control subjects increased significantly during both diets (HS: from 75.2 kg (SD 14.3) to 75.5 kg (SD 14.3), P = 0.007; LS: from 74.4 kg (SD 14.1) to 75.1 kg (SD 14.2), P < 0.001). The relative increase was higher during LS intake than during HS intake (1.1% (SD 0.5) vs. 0.4% (SD 0.6), P < 0.001).

P-Na increased in response to the hypertonic saline infusion in both groups during both diets with maximum after 60 minutes. During LS intake the relative increase in p-Na was slightly but significantly higher in the patients compared to the controls (3.6% (SD 0.9) vs. 3.0% (SD 0.9), P = 0.039). The relative increases were not significantly different between HS and LS intake in any of the two groups.

P-albumin fell significantly in response to the hypertonic saline infusion in both groups during both diets to comparable levels at the end of the study day (data not shown). The decrease was significantly higher during LS intake.

#### GFR

During LS intake, the hypertonic saline infusion caused a significant decrease in GFR in the patient group 60 minutes after infusion start (Table 6). The difference in the reductions in GFR between the two diets was not statistically significant (P = 0.688).

## Discussion

In the present study, we compared u-AQP2_CR _and u-ENaC_β-CR _in patients with essential hypertension and normotensive control subjects during HS and LS intake. Furthermore, we compared the relative change in u-AQP2_CR _and u-ENaC_β-CR _in response to a hypertonic saline infusion in patients and controls. U-AQP2_CR _and u-ENaC_β-CR _were normal in essential hypertension at baseline. However, in response to the hypertonic saline infusion, u-AQP2_CR _was abnormally increased in essential hypertension, whereas the response in u-ENaC_β-CR _was normal.

### U-ENaC_β-CR _and u-AQP2_CR _are not increased in essential hypertension at baseline

Both the systolic and the diastolic blood pressures were significantly higher in the patients than in the normotensive controls during both diets as expected. The subjects were studied in the supine position, which explains the rather low blood pressure levels in both groups throughout the study days.

Surprisingly, and in contrast with results from experimental models of hypertension [16-18,33,34] and patient with essential hypertension carrying polymorphisms of adducin [35], we found that u-ENaC_β-CR _and u-AQP2_CR _were not abnormal in patients with essential hypertension. U-osm, V, U_Na_, and FE_Na _were similar in patients with essential hypertension and normotensive controls during both HS and LS intake.

Thus, altogether these results indicate that the expression of AQP2 and the expression of ENaC are not increased in essential hypertension.

AVP and u-cAMP were not increased in the patients with essential hypertension compared to the normotensive controls during any of the diets. The activity of the renin-angiotensin-aldosterone system has previously been reported to be suppressed in hypertensive patients compared with normotensive controls during low and normal sodium intakes [36]. In the present study, Ang II was suppressed in patients compared with controls but only during HS intake. PRC and Aldo were similar in patients and controls during both diets. We cannot explain the discrepancy between the two studies. Thus, apart from Ang II, the levels of the main hormonal regulators of the expression of AQP2 and ENaC are similar in patients with essential hypertension and normotensive controls.

We found a significantly lower u-AQP2_CR _during LS intake than during HS intake in both groups and a concomitant significantly lower V during LS intake. Recently, our group reported lower AQP2 during LS intake compared to during HS intake in young healthy subject, in agreement with the present study [37]. In the previous study, we discussed the possibility that an increase in the reabsorption of water proximal in the nephron during LS intake was followed by a fine-tuning in the collecting duct principal cells with decreased expression of AQP2 [37]. In support of this, Kulick et al. measured a higher absolute fluid reabsorption in rat proximal tubule during LS intake by direct in vivo microperfusion and recollection. The responsible mechanism was the adenosine type 1 receptor [38].

One should remember that both patients and control subjects were water loaded in order to make urine collection possible. We believe that this is the reason for the low osmolalities during the first hours of the study days.

We found a significantly lower u-ENaC_β-CR _during LS intake compared to during HS intake in the patients with essential hypertension at baseline, but not in the normotensive controls. LS intake is known to increase open probability of ENaC and the number of active ENaCs in normotensive mice [39]. No difference in u-ENaC_β-CR _between HS and LS intake was present in the patients with essential hypertension during the 24-h urine collection. Therefore we speculate that the difference in u-ENaC_β-CR _between HS and LS intake at baseline in the patients with essential hypertension could be by chance.

### Abnormally higher increase in u-AQP2_CR _in response to hypertonic saline in essential hypertension

U-AQP2_CR _increased significantly more in response to the hypertonic saline infusion in patients with essential hypertension than in normotensive controls, which suggests that the patients reabsorb a larger proportion of the infused water. Thus, somehow the antidiuretic effect of the hypertonic saline is increased in essential hypertension. The underlying regulatory mechanism is unknown. Abnormalities in AVP, PRC, Ang II, Aldo, ANP, BNP, u-PGE_2_, u-cAMP, GFR, blood pressure and heart rate were not responsible for the difference, since these effect variables changed in similar manner in patients and controls.

Jackson et al. have previously shown that Ang II has an enhanced ability to reduce urinary cAMP excretion in spontaneously hypertensive rats [40]. We speculate, that the larger increase in u-AQP2 in response to the hypertonic saline in patients with essential hypertension could reflect an abnormal increase in cAMP in patients with essential hypertension in response to the fall in Ang II. Whether an abnormal sensitivity to Ang II with regard to inhibiting the adenylyl cyclase is involved in the pathogenesis of essential hypertension, will be the subject of future investigations.

To our knowledge this is the first time that the change in u-AQP2_CR _in response to a hypertonic saline infusion has been measured in patients with essential hypertension. U-AQP2_CR _increased considerably, but the regulatory mechanism is unknown.

### Normal response in u-ENaC_β-CR _to hypertonic saline in essential hypertension

One would have expected a decrease in u-ENaC_β-CR _in response to the hypertonic saline infusion, since Aldo decreased and U_Na _and FE_Na _increased in both patients and controls. Instead we found a small but significant increase in u-ENaC_β-CR _in response to the hypertonic saline. The explanation of this phenomenon is not clear for the time being. A considerable decrease in the renal sodium absorption more proximal in the nephron might be compensated for by an increase in absorption in the distal part of the nephron, but additional studies are required to determine the precise use of u-ENaC_β-CR _as biomarker for ENaC activity. Whether membrane shedding plays a role in the increase in u-ENaC awaits further studies. A significantly higher natriuretic response was observed in the patients two hours after the hypertonic saline infusion during HS intake (Table 6). The similar change in the glomerular filtered load of sodium in patients and controls suggests that a reduction in renal tubular sodium reabsorption contributes to the higher natriuretic response in patients with essential hypertension during HS intake. ENaC does not seem to be responsible for the decreased tubular sodium reabsorption, since the change in ENaC in response to the hypertonic saline infusion was similar in patients and controls. The systolic blood pressure increased significantly during HS intake in the patients, but not in the controls, and could have contributed to the higher natriuretic response. The exaggerated natriuretic response in essential hypertensive patients has been reported in numerous studies. It has been attributed both to altered handling of sodium in both proximal and distal tubules, to an exaggerated increase in ANP, and to an increased GFR, but the exact intra-renal mechanism is unknown. However, the response in u-ENaC_β-CR _to hypertonic saline infusion was the same in patients with essential hypertension and normotensive controls. Thus, an abnormal regulation of ENaC could not explain the phenomenon of exaggerated natriuresis in essential hypertension.

### Strength and limitations

The strengths of this study lie in the design. The study is a controlled clinical study with an age- and sex-matched control group. The salt intake and fluid intake are standardised and controlled. Furthermore the patients with essential hypertension pause their antihypertensive medication during the study. Thus, the results are not confounded by different salt or water balance or antihypertensive drugs.

A limitation of the study is that the threshold for hypertension is quite modest, which may explain why few differences are seen. We cannot exclude that we would have found a more pronounced difference in the AQP2 response to the hypertonic saline between patients and controls, if we had included patients with more severe hypertension.

## Conclusions

In conclusion, no difference was found in u-AQP2_CR _and u-ENaC_β-CR _between patients and controls at baseline. However, in response to the hypertonic saline infusion, u-AQP2_CR _was abnormally increased in essential hypertension, whereas the response in u-ENaC_β-CR _was normal. The underlying mechanism for the abnormal regulation of AQP2 expression is not clarified, but it is not AVP-dependent.

## Competing interests

The authors declare that they have no competing interests.

## Authors' contributions

CCG conceived of the study, participated in the design, coordination of the study, and recruitment of subjects, carried out the experiments, analyzed the data, performed the statistical analysis, and drafted the manuscript. JNB helped to draft the manuscript. TGL assisted in the experimental part of the study. HV assisted in the experimental part of the study. EBP conceived of the study, participated in the design of the study and the recruitment of subjects, was responsible for the laboratory analyses, and helped to draft the manuscript. All authors read and approved the final manuscript.

## Pre-publication history

The pre-publication history for this paper can be accessed here:

http://www.biomedcentral.com/1471-2369/13/15/prepub
